# Left ventricular twist before and after haemodialysis: an analysis using speckle-tracking echocardiography

**DOI:** 10.5830/CVJA-2018-019

**Published:** 2018

**Authors:** Yip Anthony, Peters Ferande, Libhaber Elena, Maharaj Nirvathi, Rafique Essop Mohammed, Naicker Saraladevi, Mashabane Mduduzi, Yip Anthony, Naicker Saraladevi, Peters Ferande, Libhaber Elena, Maharaj Nirvathi, Mashabane Mduduzi, Rafique Essop Mohammed

**Affiliations:** Division of Cardiology, Chris Hani Baragwanath Hospital, Johannesburg, South Africa; Division of Cardiology, Chris Hani Baragwanath Hospital, Johannesburg, South Africa; Division of Cardiology, Chris Hani Baragwanath Hospital, Johannesburg, South Africa; Division of Cardiology, Chris Hani Baragwanath Hospital, Johannesburg, South Africa; Division of Cardiology, Chris Hani Baragwanath Hospital, Johannesburg, South Africa; Department of Internal Medicine, Charlotte Maxeke Johannesburg Academic Hospital, South Africa; Division of Nephrology, Chris Hani Baragwanath Hospital, Johannesburg, South Africa; Faculty of Health Sciences, University of the Witwatersrand, Johannesburg, South Africa; Faculty of Health Sciences, University of the Witwatersrand, Johannesburg, South Africa; Faculty of Health Sciences, University of the Witwatersrand, Johannesburg, South Africa; Faculty of Health Sciences, University of the Witwatersrand, Johannesburg, South Africa; Faculty of Health Sciences, University of the Witwatersrand, Johannesburg, South Africa; Faculty of Health Sciences, University of the Witwatersrand, Johannesburg, South Africa; Faculty of Health Sciences, University of the Witwatersrand, Johannesburg, South Africa

**Keywords:** left ventricular twist, speckle-tracking echocardiography, chronic kidney disease, dialysis

## Abstract

**Background:**

The most commonly used parameter of cardiac function in the chronic kidney disease (CKD) patient is ejection fraction (EF), using transthoracic echocardiography (TTE). EF is a highly load-dependent measurement, which varies considerably in CKD patients undergoing haemodialysis. The aim of this pilot study was to evaluate a novel measure of myocardial function, left ventricular twist, which is defined as the ‘wringing action of the heart’, using speckletracking echocardiography in CKD patients before and after haemodialysis.

**Methods:**

Twenty-six patients were recruited from the Chris Hani Baragwanath Hospital haemodialysis unit. TTE was performed according to a detailed standardised protocol before and after a single haemodialysis session. Echocardiography was also performed on 26 age- and gendermatched healthy subjects.

**Results:**

The mean age of the control versus CKD group was 44 ± 11.4 and 43.4 ± 12.2 years, respectively; 46% were male. Apical rotation was diminished in CKD patients compared to controls (4.83 ± 2.3 vs 6.31 ± 1.6 °; p = 0.01) despite no difference in EF (61.7 ± 6.2 vs 58.8 ± 13; p = 0.68). There were no differences in the components of twist: apical rotation, basal rotation and net twist before and after dialysis, despite an increase in EF (58.8 ± 13.7 vs 61.2 ± 13.6; p = 0.02) following dialysis.

**Conclusion:**

Unlike EF, the components of twist are relatively independent of changes in haemodynamic load seen during dialysis. The decrease in apical rotation may represent an early marker of cardiac pathology in the late-stage CKD patient.

Cardiovascular pathology accounts for half of the deaths in chronic kidney disease (CKD) patients.^1^,^2^ Causes of increased mortality rates include sudden death from arrhythmias, heart failure and ischaemic heart disease.^1^,^3^-^6^ Transthoracic echocardiography (TTE) is the most commonly used imaging modality to assess cardiac function in patients with CKD. However, the most widely used echocardiographic measurement is ejection fraction (EF), which is load dependent and varies considerably with the volume shifts experienced during haemodialysis.^7^

Cardio-renal specialists have explored other measures to evaluate cardiac function in CKD, using myocardial deformation or strain, which more accurately describes ventricular movement during systolic and diastolic function. It consists of longitudinal, radial and circumferential strain, and ventricular twist.^8^ Tissue Doppler imaging was previously used to measure strain but required correct alignment of the Doppler signal to the angle of the myocardial fibres.^8^ In recent years, speckle-tracking echocardiography (STE) has emerged as a potentially more accurate technique to measure myocardial deformation.^9^

STE is an echocardiographic modality based on the accurate tracking of groups of pixels called ‘speckles’ throughout the cardiac cycle.^9^ Using this technique, parameters reflecting myocardial deformation, such as global longitudinal strain, radial and circumferential strain, and left ventricular twist can be assessed. The initial studies conducted in chronic kidney disease patients using speckle tracking to evaluate myocardial function demonstrated a reduction in longitudinal strain but not in circumferential or radial strain and did not evaluate left ventricular twist.^10^-^12^

Left ventricular (LV) twist is described as a ‘wringing’ action of the heart. It represents a clockwise rotation of the base and a counter-clockwise rotation of the apex during systole.^13^Using STE, LV twist^14^ has been validated against magnetic resonance imaging (MRI)^15^ and evaluated in conditions such as hypertension,^16^ ischaemic heart disease^17^ and a variety of cardiomyopathies.^18^ In CKD, LV twist has been shown to increase as calculated glomerular filtration rate (GFR) decreased.^19^-^21^ A major limitation of the above studies on LV twist is that the impact of varying loading conditions in patients undergoing haemodialysis was not evaluated.

The aim of this pilot study was to examine LV twist in African patients with stage 5 CKD before and after a single haemodialysis session.

## Methods

This prospective, longitudinal, single-centre study was conducted at the Chris Hani Baragwanath Hospital Renal Unit in Johannesburg, South Africa. Volunteers were screened from November 2010 until February 2011. Inclusion criteria were: ages between 20 and 65 years and documented CKD on intermittent haemodialysis three times weekly. Exclusion criteria were: pre-existing cardiac disease, known coronary artery disease, valvular heart disease, arrhythmias, and poor echocardiography windows that precluded speckle tracking.

**Fig. 1:**
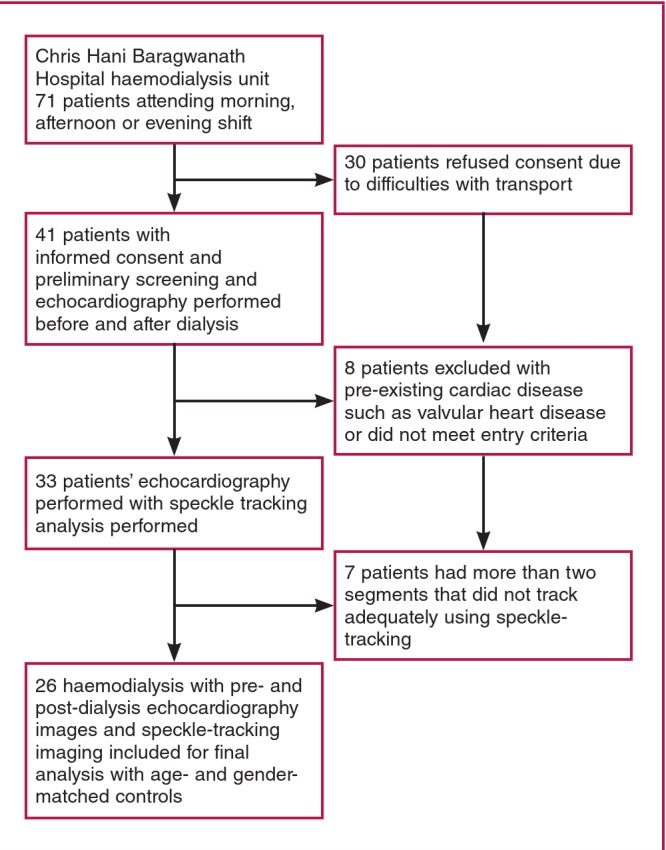
Flow chart of patient recruitment.

Of the 71 patients receiving intermittent haemodialysis in this unit, 26 meeting the entry criteria were recruited among volunteers (Fig. 1). Similarly, 26 age- and gender-matched individuals were recruited from healthy volunteers with no known underlying medical conditions among unrelated staff members at Chris Hani Baragwanath Hospital and local churches around the Soweto, Johannesburg area.^22^

**Fig. 2:**
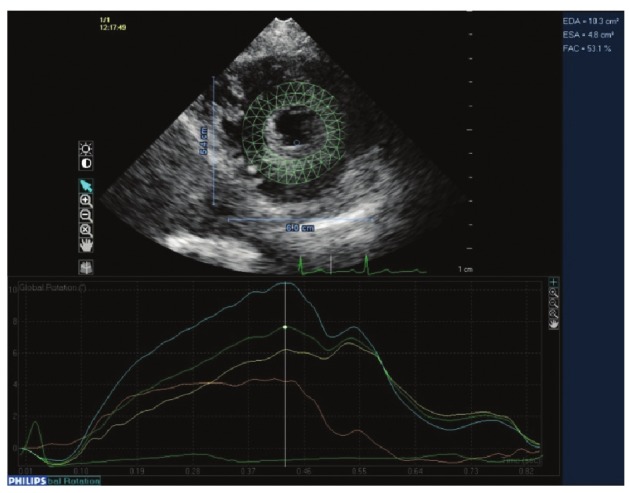
Short-axis view through the apex.

**Fig. 3:**
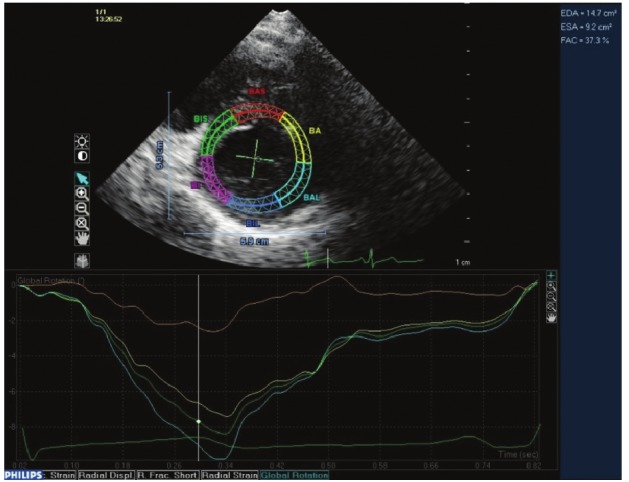
Basal rotation in the short-axis view.

Ethics approval for this study was obtained from the University of the Witwatersrand human research ethics committee. Written informed consent was obtained from all patients, and the study protocol (approval number M10510) conformed to the 1975 Declaration of Helsinki.

Patients with CKD with end-stage kidney failure (ESKF) were on three-times-a-week intermittent haemodialysis (HD). Haemodialysis was performed for an average of four hours with mean ultrafiltration volume of 2.2 ± 0.9 litres, using bicarbonate dialysate. Fresenius FX dialysers were used, with most patients dialysed on the FX 80 dialysers, although the range of dialysers used were FX 60, FX 80 and FX 100, according to the patient’s weight.

The recombinant erythropoietin, epoetin-beta, was used to maintain haemoglobin levels at a target of 11 to 12 g/dl, in keeping with KDIGO guidelines at the time. An average of 12 000 units was given subcutaneously per patient per week. Eighty-eight per cent of patients receiving haemodialysis were on an ACE inhibitor or angiotensin receptor blocker, with the most frequently used agents being perindopril and telmisartan.

All 52 participants underwent complete transthoracic echocardiographic evaluation. CKD patients were evaluated before and within an hour of a single haemodialysis session. According to a standardised protocol used by our institution,^22^-^25^ a comprehensive echocardiographic examination was performed in the lateral decubitus position using a commercially available system (iE33 xMATRIX, Philips Healthcare, Andover, MA, USA) equipped with an S5-1 transducer (frequency transmitted 1.7 MHz, frequency received 3.4 MHz). Measurements obtained were averaged from three heartbeats. All data were transferred to an Xcelera workstation (Phillips Healthcare) for offline analysis.

Chamber size measurements and function were performed according to the American Society of Echocardiography (ASE) chamber quantification guidelines of 2006.^26^,^27^ EF was calculated using LV volumes with the modified biplane Simpson’s rule, in keeping with guidelines.^26^ Diastolic function was evaluated and analysed in accordance with the ASE 2009 guidelines.^28^

Left ventricular end-diastolic volume (LVEDV) was taken as representative markers of preload. Pulse pressure over stroke volume (PP/SV) was used as a surrogate of arterial stiffness,^29^,^30^ which takes into account the contributions of systemic vascular resistance (SVR) and ventricular compliance to afterload.^31^ PP/SV has previously been validated as a measure of arterial stiffness and afterload in trials such as the LIFE study.^32^ Mean arterial pressure (MAP) was used as an indirect marker for afterload as it is a major contributor to SVR.

Speckle-tracking basal images were obtained in the parasternal short axis at the level of the mitral valve, showing the tips of leaflets with the most circular image possible. Apical images were acquired by moving the transducer one or two spaces caudally, using a method described by van Dalen.^33^

Images were acquired at a frame rate of 50–80 frames/s during sinus rhythm with less than 10% variability in heart rate for optimal speckle tracking.^18^ These images were reviewed and analysed by a cardiologist experienced in STE, using QLAB Advanced Quantification software (Version 8.0, Philips Healthcare).^9^,^34^ Tracking points were placed within the myocardium to avoid the pericardium. In keeping with ASE/European Association of Echocardiography (EAE) consensus,^35^ counter-clockwise rotation was assigned a positive value and clockwise rotation a negative value as viewed from the apex (Figs 2, 3).

Peak apical rotation was measured during the ejection systolic phase. Basal rotation was measured at a time isochronous to peak apical systolic rotation, in keeping with a standard protocol used by our institution, which has previously been published.^22^,^24^,^25^ Net instantaneous twist was calculated as peak apical rotation minus the isochronous basal rotation.

Measurements were independently made by two cardiologists trained in STE. The combined mean inter-observer variability for measurements of apical, basal and net twist of renal patients pre-dialysis was 3.67% (range 2–37%) and post-dialysis 3.7% (range 2.5–31%). The mean intra-observer variability pre-dialysis was 2.76% (range 2–10%) and post-dialysis 3.72% (range 2.5–26%).

## Statistical analysis

Data were analysed using the Statistica version 11 (Statsoft; Tulsa, Oklahoma, USA) program. Results are expressed as means with standard deviations or medians for non-normal distribution or frequencies, and percentages for categorical variables. To assess differences between the control groups versus pre-dialysis patients, and control versus post-dialysis patients, the Mann–Whitney test for non-normally distributed variables was used. Pre-dialysis and post-dialysis comparisons were performed with the Wilcoxon matched paired test. Significance was assumed at two-sided values of p < 0.05. Fisher’s exact test was used to compare categorical data. The Schapiro–Wilk test was used to assess normality. Univariate linear regression analysis was used to identify independent factors associated with twist pre-dialysis and post-dialysis, and change in twist.

## Results

Clinical characteristics of control participants and CKD patients are summarised in Table 1. The mean ages of control versus CKD patients were 44.0 ± 11.4 versus 43.4 ± 12.2 years (p = 0.81), with a 46% male incidence in both groups. The most common aetiology of the CKD patients was hypertension (81%). Weight (mean 66.2 ± 8.5 vs 65.2 ± 12.9 kg; p = 0.44), body mass index (BMI) and body surface area (BSA) were similar between the groups (Table 1). By contrast, with the CKD patients, the pre-dialysis systolic blood pressure, diastolic blood pressure, mean arterial pressure and pulse pressure were significantly higher compared to levels observed in the control group (Table 1).

**Table 1:** Clinical characteristics of patients and controls

*Characteristics*	*Control (n = 26)*	*Pre-dialysis (n = 26)*	*Post-dialysis(n = 26)*
Mean age (years)	44.0 ± 11.4	43.4 ± 12.2	–
Male gender, n (%)	12 (46)	12 (46)	–
Height (cm)	163.6 ± 8.9	164.0 ± 9.6	–
Weight (kg)	66.2 ± 8.5	65.2 ± 12.9	63.0 ± 12.6†
Change in weight (kg)	–	–	2.2 ± 1.0
Haemoglobin (g/dl)	–	9.9 ± 2.3	-
Heart rate (beats/min)	70.3 ± 11.9	81.8 ± 11.9*	89.7 ± 18.3
Body mass index (kg/m^2^)	24.7 ± 2.5	24.2 ± 4.0	–
Body surface area (m^2^)	1.7 ± 0.1	1.7 ± 0.2	–
Diabetes mellitus, n (%)	0	2 (8)*	–
Hypertension, n (%)	0	22 (81)*	–
Systolic blood pressure (mmHg)	122.7 ± 5.1	151.8 ± 17.6*	145.0 ± 24.5
Diastolic blood pressure (mmHg)	75.5± 10.2	90.1 ± 14.1*	88.4 ± 16.5
Mean arterial pressure (mmHg)	91.2 ± 7.4	110.6 ± 13.7*	107.4 ± 18.0
Pulse pressure (mmHg)	47.2 ± 10.3	61.7 ± 14.4*	56.6 ± 16.3
Volume ultra-filtrated (l)	–	–	2.2 ± 0.9
Years on dialysis	–	6.7 ± 3.4	–
Corrected calcium (mmol/l)	–	2.3 ± 0.3	–
Corrected calcium (g/dl)	–	9.2 ± 1.3	–
Phosphate (mmol/l)	–	1.3 ± 0.5	–
Phosphate (g/dl)	–	4.1 ± 1.7	–
Calcium × phosphate product (g2/dl2)	–	37.6 ± 15.5	–
Parathyroid hormone level (pg/ml)	–	66 ± 68	–

*p-value < 0.05 vs control group, †p-value < 0.05 vs pre-dialysis group.

On echocardiography, patients on haemodialysis had significantly higher pre-dialysis LV diastolic volumes, LV end-systolic volumes, LV end-systolic diameter (LVESD) and stroke volume compared to the controls, whereas there was no difference in EF and PP/SV (Table 2). In addition, patients on haemodialysis had significantly thicker LV walls and greater LV mass compared to controls. LV hypertrophy (LVH) was present in 88% of renal patients (23 of 26 patients). In those patients with hypertrophy, 96% (22 patients) were concentric in pattern and 4% had eccentric hypertrophy (one patient). As expected, patients had diastolic dysfunction with significantly greater indices of elevated filling pressure [E/E′ and left atrial (LA) volume index] pre-dialysis compared to the normal control group.

**Table 2:** Echocardiographic characteristics

*Characteristics*	*Control (n = 26)*	*Pre-dialysis (n = 26)*	*Post-dialysis (n = 26)*
LV end-diastolic volume (ml)	71.0 ± 9.8	97.9 ± 39.2*	83.5 ± 23.9†
LV end-systolic volume (ml)	30.6 ± 7.6	41.1 ± 23.7	35.2 ± 20.3†
Stroke volume (ml)	40.5 ± 10.2	57.4 ± 28.3*	49.3 ±16.5
LV end-diastolic diameter (mm)	44.9 ± 0.3	45.8 ± 0.7	45.3 ± 0.6
LV end-systolic diameter (mm)	28.8 ± 0.4	32 .0 ± 0.6*	29.7 ± 0.6
Interventricular septal diameter (mm)	10.0 ± 0.2	14.1 ± 0.3*	14.0 ± 0.3
Posterior wall thickness (mm)	9 .0 ± 0.1	13.5 ± 0.3*	13.2 ± 0.3
Relative wall thickness (mm)	0.4 ± 0.04	0.6 ± 0.1*	0.6 ± 0.1
Ejection fraction (%)	61.7 ± 6.2	58.8 ± 13.7	61.2 ± 13.6†
LV mass index (g/m^2^)	84.5 ± 18.9	156.1 ± 61.9*	152.7 ± 62
Left atrial volume index(ml)	25.8 ± 5.6	33.4 ± 15.2 *	27.8 ± 15.6†
Mitral E/A (ratio)	1.2 ± 0.4	1.1 ± 0.4	1.1 ± 0.7
E/E′ (ratio)	9.8 ± 2.4	15.2 ± 5.2*	13.0 ± 5.8†
Pulse pressure/stroke volume (mmHg/ml)	1.3 ± 0.8	1.4 ± 0.9	1.3 ± 0.8

*p-value < 0.05 vs control group, †p-value < 0.05 vs pre-dialysis group‡

During dialysis, CKD patients were ultra-filtrated a mean of 2.2 ± 0.9 litres, with a mean change in weight of 2.2 ± 1.0 kg. As a result, there was a significant difference in pre- and post-dialysis weights (Table 1). No statistically significant differences between systolic, diastolic, mean arterial pressure and heart rate were found.

There was a significant decrease in LVEDV, LVESV, E/E′ and LA volume index (LAVI) after dialysis whereas a significant increment in EF was noted compared to pre-dialysis values (Table 2). However, the stroke volume and PP/SV did not change.

At baseline, there was no difference in net speckle-tracking twist and basal rotation between controls compared to CKD patients prior to their dialysis session. However, there was a significant decrease in apical rotation between the control and pre-dialysis group (6.3 ± 1.6 vs 4.8 ± 2.3°; p = 0.01). There was no statistically significant difference when comparing net twist, basal rotation or apical rotation in CKD patients before and after dialysis (Table 3).

**Table 3:** Speckle-tracking characteristics

*Characteristics*	*Control (n = 26)*	*Pre-dialysis (n = 26)*	*Post-dialysis (n = 26)*
Apical rotation (°)	6.3 ± 1.6	4.8 ± 2.3*	5.5 ± 3.6
Basal rotation (°)	–3.3 ± 1	–3.4 ± 1.9	–3.3 ± 1.9
Net twist (°)	9.6 ± 1.9	8.2 ± 3.1	8.8 ± 4.1

*p-value < 0.05 vs control group.

In the univariate linear regression analysis of twist, the presence of hypertension, diabetes, the use of ACE inhibitor or angiotensin receptor blocker (ARB), and change in weight before and after dialysis were compared against the difference in apical, basal and net twist before and after dialysis. These variables showed a trend towards statistical significance with an independent association between hypertension and the difference in apical twist (regression coefficient of 0.34; p = 0.088), and in the use of an ACE inhibitor or ARB versus net twist (regression coefficient of 0.34; p = 0.09). A significant association was demonstrated between the differences in systolic and diastolic blood pressure versus basal twist post-dialysis (p = 0.02 and p = 0.006, respectively), and the difference in diastolic blood pressure and apical twist post-dialysis (p = 0.04).

## Discussion

The major findings of this study are (1) apical rotation appears to be reduced in patients on chronic haemodialysis with net twist remaining unchanged; and (2) LV twist is less susceptible to haemodynamic fluctuations associated with dialysis than EF.

The use of EF as a measure of systolic function in CKD is suboptimal because of the variable load changes and the effects of uraemic metabolites during dialysis. According to the ‘Starling effect’, LV function is determined by load, with increasing preload resulting in improved LV function, and vice versa. Similarly, systolic function is inversely related to afterload. However, it is not only load changes that play a role in systolic function in CKD patients on dialysis. An additional possibility is that the removal of negatively inotropic uraemic toxins during haemodialysis improves cardiac function.^7^,^11^,^36^ In clinical practice, trying to predict the relative interplay of load changes and uraemia on EF is extremely complex.^7^

In this study, CKD patients had similar EF to the control participants at baseline, which is not surprising since systolic dysfunction is seen in only 15% of CKD patients.^37^ During dialysis, there was a significant reduction in preload (LVEDV, LVESV, LAVI and E/Ea ratios), but no significant change in afterload (MAP and PP/SV ratios).^31^ Therefore, it would be reasonable to postulate that the EF should have been reduced, according to Starling. Since EF increased after dialysis, the removal of uraemic metabolites during haemodialysis may have been responsible for the improvement.^7^

Considering these changes, one might suppose that if apical, basal and net twist were subject to load changes, any or all of these parameters would decrease with reduced preload. These measures of rotation did not change with dialysis. This lack of significant change after dialysis implies that the components of myocardial rotation: apical rotation, basal rotation and net LV twist are relatively load independent, but whether they are also relatively immune to the acute metabolic changes of uraemia requires further study.

**Fig. 4:**
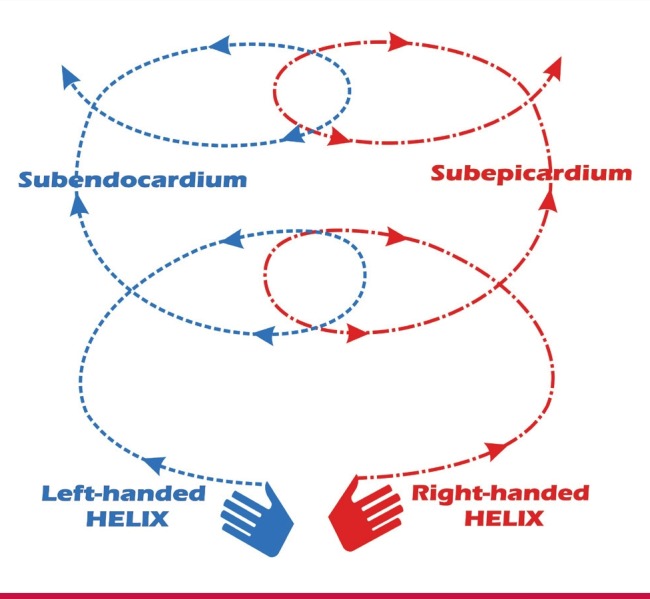
Myocardial fibre orientation and direction. Left-handed helical orientation of the sub-endocardium. Righthanded helical arrangement of the sub-epicardium.

The key to understanding LV twist and its contribution to cardiac systolic function is in understanding the arrangement of myocardial fibres in a ‘left-handed’ helix sub-endocardially with clockwise rotation, and a ‘right-handed’ helix sub-epicardially with counter-clockwise rotation (Fig. 4). In normal cardiac physiology, apical rotation provides the greater contribution to net twist because of the larger radius of rotation of its sub-epicardial predominant fibres compared to the sub-endocardial predominant base. For example, conditions that are known to affect mainly the sub-endocardial layer of the myocardium, such as hypertensive LVH,^38^ aortic stenosis,^39^ hypertrophic cardiomyopathy,^39^ amyloidosis^40^ and early myocardial ischaemia^41^ have been shown to cause apical hyper-rotation through the relatively unopposed sub-epicardial muscle fibres. This may be a compensatory function to preserve systolic function, with many of these conditions showing increase in net LV twist despite a reduction in global longitudinal strain.^22^

This compensatory increase in twist present in the CKD patient was documented in a study by Panoulas et al.^19^ This study demonstrated that twist increases while longitudinal strain decreases in CKD patients with preserved EF. This is postulated to represent an adaptive mechanism to preserve EF in the face of declining longitudinal myocardial function. The increase in twist was inversely related to worsening GFR in patients with preserved EF.^19^ By contrast, our study, which only included very late-stage CKD patients, showed significant decrease in the apical myocardial rotation with no difference in net twist found in haemodialysis patients compared to controls. This is despite there being no difference in baseline EF between the dialysis patients and controls, implying that diminution of apical rotation and a normal LV twist as opposed to an expected increase in twist may be an early indicator of further myocardial dysfunction and loss of compensatory mechanisms aimed to preserve EF (Fig. 4).

The limitations of this study are that it was a pilot study using a single vendor (Philips Healthcare). The small sample size was due to the size of the haemodialysis patient population at our institution. This did not allow adequate numbers to perform multiple linear regression analysis. The homogenous nature of our study population may not translate to other patient cohorts. It would be useful in larger studies to determine how twist is affected in CKD patients with and without hypertension. Multicentre studies with longitudinal follow up may confirm the findings of this study.

## Conclusion

LV twist and its derived rotational parameters did not change significantly post-dialysis compared to pre-dialysis. This may suggest that these parameters are less affected by varying loading conditions post-dialysis. The decrease in apical rotation observed in late-stage CKD patients compared to controls may represent an early marker of loss of rotational compensation, which preserves EF in the CKD patient
